# Targeting oral tumor microenvironment for effective therapy

**DOI:** 10.1186/s12935-023-02943-5

**Published:** 2023-05-23

**Authors:** Hendrik Setia Budi, Bagher Farhood

**Affiliations:** 1 Department of Oral Biology, Dental Pharmacology, Faculty of Dental Medicine, Universitas Airlangga, Surabaya, Indonesia; 2 Department of Medical Physics and Radiology, Faculty of Paramedical Sciences, Kashan University of Medical Sciences, Kashan, Iran

**Keywords:** Oral Cancer, Tumor Microenvironment, Immunotherapy, Cancer-Associated fibroblasts, Tumor-Associated Macrophages, CD8 + T lymphocytes

## Abstract

Oral cancers are among the common head and neck malignancies. Different anticancer therapy modalities such as chemotherapy, immunotherapy, radiation therapy, and also targeted molecular therapy may be prescribed for targeting oral malignancies. Traditionally, it has been assumed that targeting malignant cells alone by anticancer modalities such as chemotherapy and radiotherapy suppresses tumor growth. In the last decade, a large number of experiments have confirmed the pivotal role of other cells and secreted molecules in the tumor microenvironment (TME) on tumor progression. Extracellular matrix and immunosuppressive cells such as tumor-associated macrophages, myeloid-derived suppressor cells (MDSCs), cancer-associated fibroblasts (CAFs), and regulatory T cells (Tregs) play key roles in the progression of tumors like oral cancers and resistance to therapy. On the other hand, infiltrated CD4 + and CD8 + T lymphocytes, and natural killer (NK) cells are key anti-tumor cells that suppress the proliferation of malignant cells. Modulation of extracellular matrix and immunosuppressive cells, and also stimulation of anticancer immunity have been suggested to treat oral malignancies more effectively. Furthermore, the administration of some adjuvants or combination therapy modalities may suppress oral malignancies more effectively. In this review, we discuss various interactions between oral cancer cells and TME. Furthermore, we also review the basic mechanisms within oral TME that may cause resistance to therapy. Potential targets and approaches for overcoming the resistance of oral cancers to various anticancer modalities will also be reviewed. The findings for targeting cells and potential therapeutic targets in clinical studies will also be reviewed.

## Introduction

The incidence of cancer is increasing in the world. Oral cavity malignancies are the 16th most common type of cancer in the world. However, the incidence of these cancers is very different for men and women [1]. GLOBOCAN 2020 reported that the incidence of oral cancers is more than 377,000 cases worldwide with more than 177,000 deaths [2]. The incidence of oral cancers is more common in developing countries like India, Bangladesh, Taiwan, and others. It has been reported that some risk factors such as smoking, human papillomavirus (HPV), alcohol, poor oral cavity hygiene, and drinking hot liquids (like tea) increase the risk of oral malignancies [3, 4]. The roles of these factors in the mutations and genomic instability, invasion, epithelial-mesenchymal transition, progression of tumors, and metastasis have been confirmed [5]. The increasing rate of risk factors and cancer incidence can enhance the demand for efficient therapy modalities.

Cancer therapy is one of the most challenging issues in medicine. To date, no perfect treatment modality or drug has been approved for the treatment of cancers [6]. The response of each cancer to therapy is different compared to other cancers. Furthermore, each tumor type consists of a wide range of malignant and non-malignant cells with different percentages compared to other tumors. The components of each type of tumor also may be different for various people with cancer. These complexities can make the therapy of tumors a big challenge [7]. Usually, oncologists suggest a combination of different modalities and anticancer drugs for more effective cancer therapy. However, this may be associated with some severe side effects that need to be considered [8, 9]. Therefore, a deep knowledge about the type of malignancies, pathogenesis, and various effective factors in the proliferation and suppression of malignant cells is crucial.

Considering the tumor microenvironment (TME) is one of the intriguing approaches for the treatment of various solid tumors. In addition to cancer cells, TME includes various non-cancerous cells that provide various signals and growth factors for the proliferation of cancer cells. Cancer cells and tumor stroma release several factors to attract immunosuppressive and pro-tumor cells. Stromal and cancer cells can induce the infiltration of myeloid-derived suppressor cells (MDSCs), tumor-associated macrophages (TAMs), and regulatory T cells (Tregs) into the tumor. These cells suppress the activity of anti-tumor immune cells such as natural killer (NK) cells and CD8 + T lymphocytes. The released cytokines, chemokines, exosomes, and other molecules by these cells affect the proliferation and also the response of cancer cells to therapy [10]. Modulation of these cells and interactions within TME is an intriguing idea for the elimination of cancer cells and enhancing the efficacy of anticancer drugs or radiotherapy [11–13].

This review explains the interactions of various components of TME with oral cancer cells. Furthermore, interesting targets and agents for overcoming oral cancer resistance regarding TME will be proposed. We also will discuss findings from clinical studies for targeting TME components on the response of oral cancers to anticancer therapy.

## Oral cancer: definition, therapies, and limitation

Oral cancer refers to any malignancy in the oral cavity. It can occur in the lips, tongue, gums, roof, and floor of the mouth, and also inner layers of cheeks [14]. Oral cancers are classified as the most abundant type of head and neck malignancies. It has been uncovered that most types of oral cancer cells are squamous cell carcinoma (SCC). Usually, this type of oral cancer is flat and may occur on the lips or within the mouth. Some other types of oral cancers are containing melanoma, sarcomas, lymphoma, polymorphous low-grade adenocarcinoma and adenoid cystic carcinoma (which may occur in salivary glands), and some others [15].

Oral cancers may need similar treatment protocols. Chemotherapy, radiotherapy, immunotherapy, and surgery may be prescribed for these malignancies, depending on the location, type, and stage of the tumor. Radiotherapy and chemotherapy are the most common anticancer modalities for most malignancies, including oral cancers. Radiotherapy uses X-rays to destroy cancer cells through the generation of a heavy amount of ROS and also by direct interactions with vital targets in the cells such as DNA [16]. Severe damage to DNA and other vital organelles such as mitochondria and endoplasmic reticulum can stimulate cell death through apoptosis, necrosis, and other cell death mechanisms. Chemotherapy drugs such as cisplatin, 5-Fluorouracil (5-FU) and docetaxel also kill oral malignant cells through different mechanisms such as inducing DNA damage, inhibiting DNA repair, and also by stimulation of tumor suppressor genes like p53 and cell cycle arrest [17–19]. Some patients may undergo a combination of chemotherapy and radiotherapy for better control of tumors and more efficient elimination of cancer cells [20].

Although chemotherapy and radiotherapy can eliminate most cancer cells, the remained cells can cause tumor recurrence and also cause resistance of cancers to therapy [21]. For example, cancer cells may develop some mechanisms to boost DNA repair responses and also resist severe oxidative stress by inducing the activity of antioxidant defense system [22, 23]. Furthermore, the overexpression of anti-apoptosis genes and epithelial-mesenchymal transition (EMT) transcription factors are involved in the resistance of oral cancers to chemotherapy and radiotherapy [24]. The resistance of oral tumors to radiotherapy and chemotherapy increases the need to further therapies, which leads to more side effects for normal tissues [25]. Therapy with radiotherapy and chemotherapy can induce severe side effects such as mucositis, xerostomia, ulcer, and dry mouth in the oral cavity. These side effects can occur following severe damage to glands, inflammation, and ulcer, leading to some problems in swallowing, talking, and breathing [26]. Treatment with a combination of chemotherapy drugs and radiotherapy may also increase the probability and severity of these side effects [27]. These side effects reduce the quality of life for patients with oral cancers. Selective targeting of cells and interactions with oral tumors may be a better strategy for the suppression of tumor growth and also the management of side effects for normal tissues. Therefore, a knowledge of TME and its therapeutic targets is crucial for the better response of tumors to therapy with lesser toxicity.

In the next section, we will explain the cells and releases in TME of solid cancers like oral malignancies. We will also discuss the interaction between the TME with oral cancer cells, which play a pivotal role in tumor growth and resistance to therapy.

## TME and interactions with cancer cells

TME comprises diverse cells with distinct secreted molecules. Cells in TME are surrounded by an extracellular matrix. Cells and their released molecules maintain the nutrition and other factors for the proliferation of cancer cells [28]. Indeed, cancer cells can secrete some chemokines and exosomes to stimulate the recruitment of immunosuppressive cells. These cells maintain the growth factors and also protect cancer cells against attacks by the immune system [29]. It has been approved that the release of some chemokines such as CCL2 by cancer and stromal cells participates in the recruitment of monocytes [30]. These cells can be polarized into tumor-associated macrophages (TAMs) [31]. Furthermore, recruitment of myeloid cells, bone marrow-derived stem cells, fibroblasts, neutrophils, naive T cells, etc., into tumors can induce polarization of these cells into myeloid-derived suppressor cells (MDSCs), CAFs, Tregs, tumor-associated neutrophils (TANs), and some others [32, 33]. These cells have several positive cross-talks with each other and also with cancer cells [34]. Cancer cells can release immunosuppressive molecules, exosomes, and chemokines to trigger the activity of immunosuppressive cells [35]. Th2 cytokines trigger the differentiation of CD4 + T cells into Tregs [36]. Furthermore, Th2 cytokines stimulate the activity and proliferation of TAMs, CAFs, MDSCs, and cancer cells [37]. In addition to suppression of anticancer immunity, these cells facilitate tumor growth by releasing pro-angiogenesis molecules, leading to the proliferation of endothelial cells and tube formation at the end of tumor vessels [38].

Anticancer immunity in tumors is consisting CD8 + T lymphocytes and natural killer (NK) cells. The release of T helper 1 (Th1) cytokines by CD4 + Th1 lymphocytes can stimulate the proliferation of NK cells and CD8 + T lymphocytes. NK cells belong to innate immunity that can detect and kill cancer cells through the detection of abnormal expression of some antigens. However, CD8 + T lymphocytes fight against cancer cells after recognizing cancer antigens by antigen-presenting cells (APCs). Detection of cancer antigens by APCs leads to an adaptive immune response by CD8 + T lymphocytes against cancer cells. Both NK cells and CD8 + T lymphocytes can kill cancer cells by releasing some enzymes such as Granzyme B and perforin. Perforin can destroy the membrane of targeted cancer cells. Granzyme B can also induce apoptosis in the presence of perforin [39]. NK cells and CD8 + T lymphocytes can also induce apoptosis in cancer cells by releasing Fas ligand (FasL), tumor necrosis factor (TNF)-α and interferon (IFN)-γ.

In summary, NK cells and CD8 + T lymphocytes are the most important anticancer immune cells in TME that can be activated following the release of Th1 cytokines. However, cancer and stromal cells can suppress the activity of these cells by releasing immunosuppressive molecules and also triggering the recruitment of other immunosuppressive cells, including MDSCs, Tregs, and TAMs [12]. These immunosuppressive cells can also inhibit the function of NK cells and CD8 + T lymphocytes by expressing co-inhibitory molecules, exosomes, and immunosuppressive cytokines [40]. The modulation of the released cytokines and other interactions in TME for boosting NK cells and CD8 + T lymphocytes can eliminate cancer cells and suppress tumor growth [41, 42].

### Role of cancer-associated fibroblasts in the progression of oral cancer

CAFs are the most prominent non-immune cells in TME [43]. CAFs are also the main player in the progression and development of tumor stroma [44]. CAFs play a pivotal role in the proliferation of cancer cells and tumor resistance to therapy. A large number of results in the experimental and clinical investigations have confirmed the key roles of CAFs in oral cancers [45]. It seems that CAFs participate in the proliferation, immune system exhaustion, and invasion of oral cancers. The release of some molecules such as TGF-β not only stimulates the proliferation of cancer cells directly but also attenuates the anticancer immunity [46]. The release of some micro RNAs (miRNAs) and long non-coding RNAs (lncRNAs) by CAFs participate in the proliferation of oral cancer cells and tumor growth. A study by Yang et al. showed a key role of lncRNA H19 in the metabolism of CAFs and the growth of oral cancer. They demonstrated that the lncRNA H19/miR-675-5p pathway in CAFs induces glycolysis, which causes the growth of oral squamous cell carcinoma (OSCC) [47]. LncRNA-CAF which specifically is upregulated in CAFs has a role in the activation of CAFs. Upregulation of lncRNA-CAF is associated with expression and the release of IL-33, leading to the proliferation of OSCC cells. OSCC cells have a positive cross-talk with CAFs through the mentioned pathway. Cancer cells can stimulate the expression of lncRNA-CAF in CAFs, which in turn enhances the release of IL-33 and the proliferation of cancer cells [48]. A reduction of the expression of some miRNAs such as miR-124 in CAFs may be involved in the proliferation of oral cancer cells [49].

CAFs participate in immune system exhaustion. These cells secrete some immunosuppressive molecules such as immune checkpoints and pro-tumor cytokines. Programmed death ligand 1 (PD-L1) is a well-known immune checkpoint that can be secreted by CAFs [50]. Furthermore, CAFs stimulate the proliferation of cancer cells and suppress the proliferation of NK cells and CD8 + T lymphocytes by releasing transforming growth factor-β (TGF-β), IL-10, IL-1, cyclooxygenase-2 (COX-2), prostaglandin E2 (PGE2), and some others [51]. The positive cross-talk of CAFs with cancer cells and also other tumor-promoting cells in TME has a key role in the suppression of the immune system in the tumor. The chemokine (C-C motif) ligand 7 (CCL7) is one of the released molecules by CAFs. CCL7 not only stimulates the proliferation and invasion of oral SCC cells [52] but also promotes the accumulation of TAMs in oral tumors [53]. The accumulation of TAMs following interactions with CAFs can exhaust the anti-tumor function of NK and CD8 + T cells by secreting TGF-β, COX-2, PGE2, IL-10, IL-4, IL-13, and some others [10]. Overexpression of these molecules in oral cancer can predict invasion, tumor growth, and resistance to therapy [54, 55].

CAFs play a pivotal role in the invasion of oral cancers. A clinical investigation evaluated the relationship of CAFs with the survival of patients with OSCC. It showed that overexpression of stromal α-smooth muscle actin (α-SMA) in cancers can predict a higher risk for early death in patients [56]. It has also been suggested that a higher expression of CAFs markers such as α-SMA is associated with higher stages of oral tumors, invasion, and metastasis [57]. CAFs are key players in tumor stiffness and facilitate the degradation of the extracellular matrix to provide invasion and metastasis for cancer cells. CAFs release a wide range of pro-fibrosis molecules such as collagen, fibrin, and fibronectin, which promote fibrosis and tumor stiffness [58]. Additionally, the release of pro-angiogenesis molecules such as vascular endothelial growth factor (VEGF) and hypoxia-inducible factors (HIFs) by CAFs increases tumor growth [59]. A study by Gaggioli et al. suggested that CAFs can generate some tracks for oral cancers in the extracellular matrix, thereby helping the invasion of OSCC. This was associated with the upregulation of the Rho/ROCK (Rho-associated protein kinase) pathway in fibroblasts [60]. The Rho/ROCK signaling pathway participates in the development of extracellular matrix and differentiation of fibroblasts into myofibroblasts, leading to fibrosis and tumor stiffness [61]. Both stiffness and degradation of extracellular matrix in tumors are in favor of tumor growth and invasion. Tumor stiffness prevents the penetration of drugs and limits the recruitment of anticancer immune cells into the tumor. However, extracellular matrix degradation reduces the pressure of the tumor to facilitate the motility and invasion of cancer cells [62]. Another study by Chen et al. confirmed the role of extracellular matrix degradation in the invasion of oral cancer cells. They showed that interactions of oral cancers and fibroblasts provide invasion of cancer by reducing the density of the extracellular matrix [63].

CAFs release some other molecules that participate in the metastasis of oral cancer cells. Hyaluronan synthetase 2 (HAS2) is one of the major released molecules by CAFs in OSCC. HAS2 can regulate the expression of matrix metalloproteinases (MMPs). It can enhance the regulation of MMP-1 while reducing the expression of its inhibitor, tissue inhibitors of metalloproteinases (TIMPs) in oral cancers. The release of HAS2 by CAFs also triggers the proliferation of cancer cells in response to TGF-β, another secretion by CAFs and other immunosuppressive cells [64]. HAS2 is a marker of invasion for OSCC and is associated with lymph node metastases [65]. It has been suggested that CAFs are leading cells in oral cancer metastasis. Indeed, CAFs may migrate to bones before the migration of oral cancer cells. An experiment by Elmusrati et al. showed that α-SMA-positive CAFs can be infiltrated into the bone and then facilitate the migration of OSCC to bone [66]. CAFs can also modulate the invasion of oral cancer cells by regulating epigenetics modulators. The release of exosomal miR-382-5p and miR-34a-5p, and also lncRNAs by CAFs stimulates the invasion of OSCC [67–70]. On the other hand, CAFs can stimulate invasion and metastasis of OSCC by inhibiting some miRNAs such as miR-34a-5p [69]. Another important target of CAFs in OSCC is miR-14. CAFs can suppress miR-14 by releasing a lncRNA, which is known as TIRY. The release of exosomes by CAFs consisting of TIRY can decrease the regulation of miR-14 in OSCC, which lead to upregulation of the Wnt/β-catenin signaling pathway, leading to EMT, invasion, and metastasis [71]. CAFs can produce lactate in the tumor. Accumulation of lactate is associated with tumor acidity, which exhausts CD8 + T lymphocytes and triggers the proliferation of oral cancer cells [72].

**Fig. 1:**
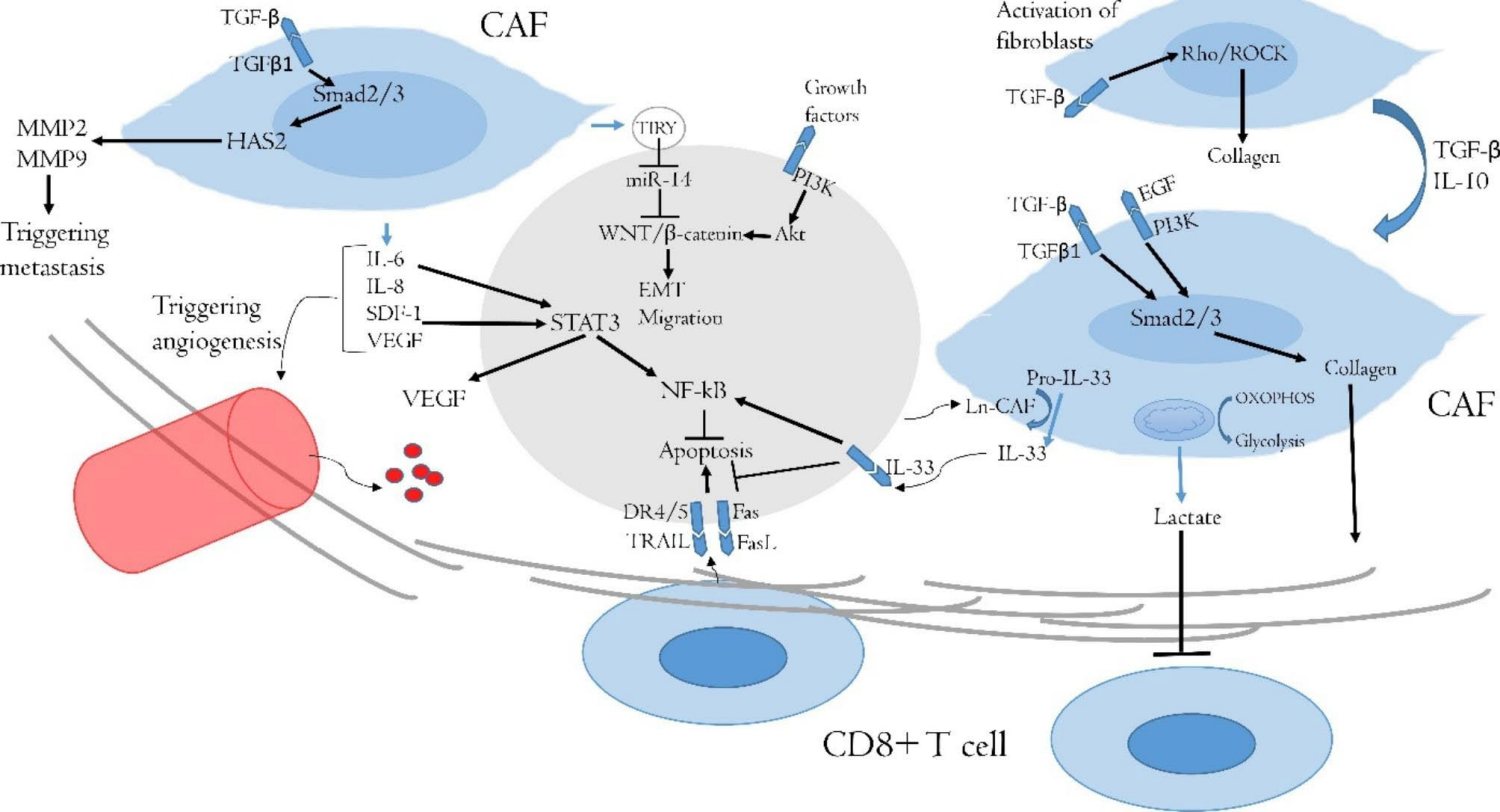
The interactions of CAFs with OSCC. CAFs have positive cross-talk with OSCC cells. Deposition of collagen and also the release of lactate acid prevent penetration of CD8 + T cells into the tumor and exhaust the activity of infiltrated CD8 + T cells. OSCC cells trigger the release of IL-33 by CAFs, leading to the inhibition of apoptosis signaling pathways in OSCC cells. The release of some other cytokines may cause overexpression of VEGF and the induction of angiogenesis. Furthermore, overexpression of HAS2 in CAFs and stimulation of the Wnt/β-catenin in OSCC cells facilitate invasion and metastasis. (CAF: Cancer-associated fibroblast; DR: Dead receptor; EGF: Epidermal growth factor; EGFR: Epidermal growth factor receptor; EMT: epithelial-mesenchymal transition; HAS2: Hyaluronan synthetase 2; TGF-β: transforming growth factor-β; MMP: Matrix metalloproteinase; NF-κB: nuclear factor-κB; PI3K: Phosphoinositide 3-kinase; SDF-1: Stromal cell-derived factor-1; STAT: Signal transducer and activator of transcription; VEGF: Vascular endothelial growth factor)

It seems that targeting of CAFs in oral cancer therapy needs to be considered. CAFs participate in the progression and resistance to therapy for a wide range of solid tumors. Furthermore, CAFs enhance resistance to various anticancer modalities such as immunotherapy, chemotherapy, and radiotherapy [7]. CAFs can resist massive oxidative stress following radiotherapy or chemotherapy. This issue may cause more accumulation of CAFs compared to NK and CD8 + T cells following exposure of the tumor to radiotherapy [73]. NK and CD8 + T cells are more sensitive to oxidative stress and DNA damage. In addition, the release of tumor-promoting molecules by CAFs may be increased after radiotherapy or chemotherapy. It has been reported that DNA damage in CAFs following exposure to radiation can induce more expression of TGF-β by CAFs, which leads to stimulation of invasion in OSCC [74]. An experiment by Liu et al. uncovered that irradiation of CAFs stimulates the proliferation, invasion, and survival of Cal-27 cells. Interestingly, irradiation of CAFs leads to some changes in the biological behavior of CAFs, which could improve DNA repair of cancer cells and enhance resistance to ionizing radiation [75]. Similar results have been revealed following the treatment of OSCC and CAFs with cisplatin or 5-FU [76–78]. A clinical study investigated the infiltration of CAFs in OSCC and its correlation with the survival of patients that underwent chemotherapy and radiotherapy. The results confirmed that a higher number of CAFs is associated with poor survival in patients [76]. Some limited studies have assessed the targeting of CAFs for improving the response of tumors to chemotherapy or radiotherapy [79, 80]. However, it needs further studies for oral tumors. The nanodrug delivery system can improve the penetration of anticancer drugs into the extracellular matrix and attenuate positive cross-talk between CAFs and malignant cells [81]. Furthermore, targeting of CAFs is a new window for effective immunotherapy for various types of solid tumors like oral cancers [82]. (Fig. 1)

### Macrophages and oral cancers

TAMs are known as potent anticancer immune system inhibitors in various solid tumors [83]. Furthermore, these cells promote angiogenesis, the proliferation of cancer cells, and metastasis, and also boost resistance to different modalities of cancer therapy [84]. It has been uncovered that the circulating Ly6C^+^CCR2^+^ monocytes are the main origin of TAMs. These cells can be derived from hematopoietic stem cells [85]. It seems that macrophages have anti-tumor activity during recruitment into oral tumors. This indicates an M1-like phenotype of macrophages during recruitment or immediately after penetration into oral tumors. These cells express STAT-1 and IFN-γ genes in oral premalignant lesions [86]. However, exposure to released cytokines and growth factors within TME induces polarization toward M2 macrophages and TAMs. The interactions of different subsets of TAMs in oral cancers are complicated. Although M1 macrophages are known as tumor-suppressive cells, the function of M1-like TAMs may be affected by the released exosomes from oral cancers. In this condition, these M1-like TAMs can induce the migration of OSCC cells [87]. The release of IL-6 by M1-like TAMs can also trigger EMT in OSCC cells [88]. These effects show a high plasticity of macrophages in oral tumors that can suppress or trigger the progression and invasion of cancer cells. Some different subsets of TAMs including CD163^+^, CD204^+^, and CD206^+^ cells have been detected in OSCC.

The detailed mechanisms for the recruitment of monocytes into oral tumors need to be determined. However, the roles of several cytokines, exosomes, and growth factors in the polarization of macrophages and the activity of TAMs in OSCCs have been uncovered by several experimental studies. However, some studies have suggested the role of some chemokines in the recruitment of monocytes into oral cancers. For example, most oral tumors have a high expression of IL-1β. This cytokine can induce the expression of CXCR4 ligands such as stromal cell-derived factor-1 alpha (SDF-1α) [89]. CXCR4 expression can trigger the recruitment of monocytes into tumors. It has been revealed that the C-X-C motif chemokine ligand 12 (CXCL12)/CXCR4 signaling in CAFs participates in the recruitment and accumulation of TAMs in OSCC [90]. The role of these interactions between cancer cells and stroma on the infiltration of TAMs has been demonstrated. The released molecules by different cells in tumor stroma trigger infiltration of monocytes, CD11b^+^ and CD163^+^ TAMs into the OSCC tumor. Microarray analysis showed that the release of CXCL12, IL-1, IL-6, and bone morphogenetic protein 4 (BMP4) by tumor stroma participates in the recruitment of monocytes and TAMs into OSCC tumors [91]. CCL2/CCR2 and intercellular adhesion molecule-1 (ICAM-1) are other important pathways that stimulate the recruitment of TAMs into oral cancers [92, 93].

The released molecules by OSCC cells play a key role in the polarization of monocytes toward TAMs. For example, oral cancer cells can induce polarization of monocytes towards CD206^+^ cells by releasing IL-8 and plasminogen activator inhibitor-1 (PAI-1) [94]. Some other cytokines in TME stimulate polarization toward TAMs. OSCC cells can stimulate this process through the release of IL-1, IL-6, and also growth arrest-specific 6 (Gas6). TAMs and other tumor-promoting cells in TME can also facilitate the polarization of monocytes toward TAMs by releasing IL-4, IL-10, IL-13, and TGF-β. The release of these molecules can change the expression of some key genes and transcription factors in monocytes and macrophages. These changes can overexpress TAMs markers. IL-4 induces the regulation of arginase-1, a key marker of TAMs [95]. It has also been uncovered that overexpression of phosphoinositide 3-kinase (PI3K)/Akt, Gas6/Axl signaling, and also NF-κB transcription factor in oral cancer-derived macrophages are involved in polarization toward TAMs [96]. These factors can be induced by the mentioned released molecules.

TAMs participate in several functions and interactions in oral cancers. After the recruitment of monocytes and polarization toward TAMs, these cells can enhance the recruitment of more monocytes and facilitate the polarization of monocytes and M1 macrophages toward TAMs by releasing Th2 cytokines and some chemokines like CCL2 [97]. TAMs have positive cross-talk with cancer and other tumor-promoting cells like CAFs, MDSCs, TANs, and Tregs [98]. TAMs can also suppress anticancer immunity in various tumors like oral malignancies. It has been displayed that CD163^+^CD204^+^ TAMs in oral cancer suppress the anticancer potency of T lymphocytes and participate in the invasion and metastasis of cancer cells. CD163^+^CD204^+^ TAMs release PD-L1 and IL-10, leading to the exhaustion of CD3 + T lymphocytes. This subset of lymphocytes plays a pivotal role in the recognition of cancer antigens and activation of CTLs against cancers, which can be used as a prognosis factor for the survival of patients with cancer [99]. Co-culture of CD163^+^CD204^+^ TAMs with CD3 + T lymphocytes leads to suppression of the activity of CD3 + T lymphocytes. Examination of clinicopathologic factors among patients with OSCC showed that accumulation of CD163^+^CD204^+^ TAMs is associated with more suppression of anticancer immunity, and lower response to therapy and survival ratio [100]. A study showed the key role of released molecules by oral cancer cells in the polarization of macrophages and suppression of T lymphocytes. The released small extracellular by OSCC stimulated polarization toward M2 macrophages. In addition, these vesicles suppressed the expression of IFN-γ by CD4 + T lymphocytes and attenuated the proliferation of CD4 + T lymphocytes [101].

**Fig. 2:**
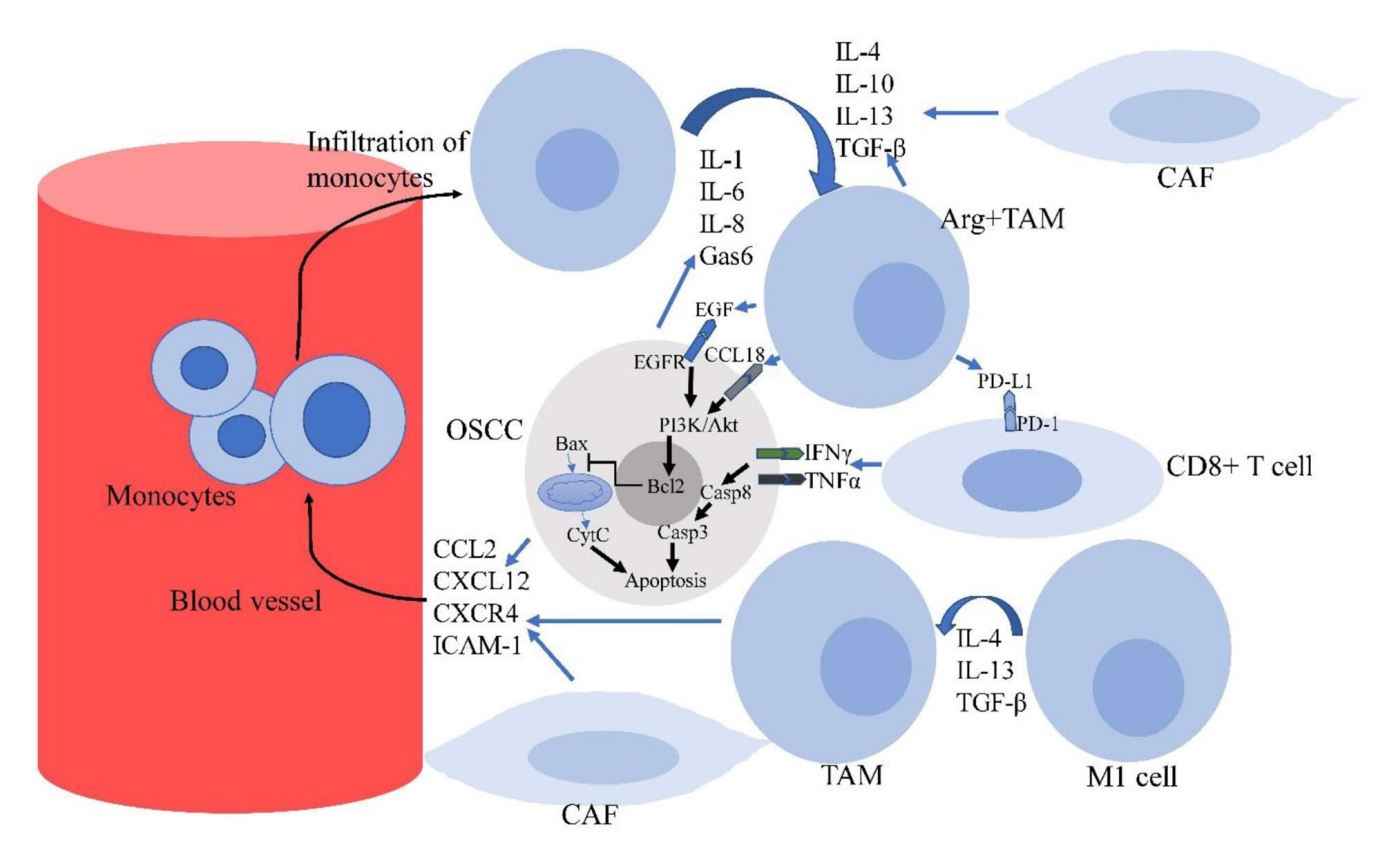
OSCC cells, CAFs, and also resident macrophages in TME release some factors such as CCL2, CXCR4, CXCL12, and ICAM-1, which lead to the recruitment of monocytes into the tumor. On the other hand, the release of IL-1, IL-6, IL-8, Gas6, IL-4, IL-10, IL-13, and TGF-β by OSCC cells, CAFs and TAMs stimulates the polarization of monocytes and alternative M1 macrophages toward TAMs. TAMs exhaust the anticancer activity of CD8 + T lymphocytes by releasing immune checkpoints like PD-L1. TAMs also stimulate the proliferation of OSCC cells by releasing EGF and CCL18. These molecules can inhibit apoptosis signaling pathways in OSCC cells. (ARG: Arginase-I; Casp: Caspase; CCR2: C-C Motif Chemokine Receptor 2; CCL-2: C-C Motif Chemokine Ligand 2; CSF-1: Colony-stimulating factor-1; CXCL12: C-X-C motif chemokine ligand 12; CXCR4: C-X-C chemokine receptor type 4; CytC: Cytochrome C; EGF: Epidermal growth factor; EGFR: Epidermal growth factor receptor; ICAM-1: Intercellular adhesion molecule-1; Gas6: Growth arrest-specific 6; GM-CSF: Granulocyte-macrophage colony-stimulating factor; TAM: Tumor-associated macrophage; TGF-β: Transforming growth factor-β; OSCC: Oral Squamous Cell Carcinoma; PD-1: Programmed death 1; PD-L1: Programmed death ligand 1; PI3K: Phosphoinositide 3-kinase; STAT: Signal transducer and activator of transcription)

The proliferation of oral cancer cells by releasing some growth factors is another important effect of TAMs in oral tumors. Epidermal growth factor (EGF) is one of the important released molecules by TAMs. A study showed that CD206^+^ TAMs can strongly produce EGF compared to other subsets of TAMs in OSCC. CD206^+^ TAMs could trigger the proliferation of OSCC cells, however, treatment with an inhibitor of EGFR suppressed the proliferation of cancer cells. This study confirmed that the release of EGF by CD206^+^ TAMs plays a key role in the proliferation of OSCC cells [102]. CCL18 is another secretion of TAMs that boosts the proliferation of cancer cells [103]. It seems that CCL18 can induce the proliferation of OSCC cells through upregulation of the PI3K/Akt pathway [104]. (Fig. 2)

Accumulation of TAMs is associated with more invasion, angiogenesis, and metastasis of oral cancers. Experimental studies and biopsies from patients can confirm this issue. A positive correlation between CD68^+^ TAMs and the density of microvessels in OSCC and oral verrucous carcinoma (OVC) has been detected [105]. The abundance of TAMs in the tumor correlates with the expression of VEGF and metastasis in patients with oral cancers [106]. TAMs play a pivotal role in the release of VEGF in hypoxic TME. This function of TAMs can be triggered following overexpression of HIF-1α [107]. The release of some other factors such as CCL13 by TAMs stimulates metastasis in oral cancers [108]. Infiltration of TAMs is also associated with poor response to therapy and worsened survival in patients with oral tumors. A clinical investigation showed that more accumulation of CD163^+^ TAMs is associated with higher grade and worsened disease-free survival for oral tongue SCC [109]. Similar findings were reported by Mori et al. [110]. Another study showed that the micro-localization of CD68^+^ TAMs in OSCC is associated with decreased T lymphocytes and poor response to therapy [111]. It seems that both CD68^+^ and CD163^+^ TAMs are involved in the invasion of OSCC and poorer survival. A study analyzed tumor species from patients with OSCC confirmed the key roles of CD68^+^ and CD163^+^ TAMs in the progression and poor prognosis of tumors. They also showed that the higher expression of TAMs markers is associated with more expression of cancer stem cells. This may indicate that TAMs can increase the invasion of oral tumors by boosting stemness in cancer cells [112].

A high number of TAMs in oral cancers is associated with resistance to chemotherapy and radiotherapy. Therefore, targeting TAMs has been suggested to reduce resistance to radiotherapy, chemotherapy, and immunotherapy for a wide range of malignancies [113]. TAMs participate in oral cancer relapse following irradiation. TAMs promote tumor recurrence by inducing neovasculogenesis [114]. It has been reported that suppression of macrophages in oral cancers enhances apoptosis in cancer cells and reduces the density of vessels within the tumor [115]. Depletion of TAMs in solid tumors like oral malignancies can remarkably increase the anticancer efficacy of radiotherapy, chemotherapy, and immunotherapy. Administration of colony-stimulating factor 1 (CSF-1) antibody is an intriguing approach for the depletion of TAMs in solid tumors. It has been shown that the administration of CSF-1 antibody suppresses TAMs in cancers and reduces resistance to therapy for various malignancies [116–118]. Suppression of chemoattracts such as CCL2/CCR2 and EGFR may be intriguing for oral cancer therapy [119, 120]. However, the efficacy of the inhibition of these targets in combination with other anticancer therapy modalities needs to be evaluated for oral cancers. Reprogramming of TAMs by some adjuvants such as nanodrugs is another interesting approach that can be suggested for overcoming the resistance of oral tumors to therapy [121]. A shift in cytokine profile in the tumor can facilitate reprogramming TAMs and M2 macrophages toward M1 macrophages. Some adjuvants like natural products and also boosting the release of IFN-γ by some drugs may be useful for reprogramming TAMs [41, 122–124]. Regorafenib is a multi-kinase inhibitor that can be used for some malignancies. It can be prescribed for the inhibition of angiogenesis by targeting VEGFR2 and also some other kinases [125]. A study suggested that regorafenib can sensitize OSCC to immunotherapy by reprogramming TAMs. Chiang et al. showed that regorafenib stimulates reprogramming TAMs toward M1 alternative macrophages in OSCC tumors, which leads to the activation of CTLs. The results also indicated that regorafenib improves the response of OSCC tumors to anti-PD-1 therapy by reprogramming TAMs. These changes were associated with the attenuation of other immunosuppressive cells such as MDSCs and Tregs [126].

### Myeloid-derived suppressor cells and oral cancers

MDSCs are a subset of immunosuppressive cells that are known as immature myeloid cells. These cells were first identified as CD34 + immunosuppressor cells in patients with head and neck cancer [127]. The secretion of cancer-derived factors stimulates the expansion of MDSCs in the bone marrow and spleen. Similar to TAMs, these cells have also potent immunosuppressive effects by overexpression of arginase-1 (Arg-1). MDSCs can also express nitric oxide synthase (NOS2), a key regulator of immune system suppression. These cells can also promote angiogenesis by releasing VEGF [128]. MDSCs have positive cross-talk with oral cancer cells and also with other tumor-promoting cells such as TAMs and CAFs [129]. The number of MDSCs in OSCC correlates with the tumor stage [130]. Furthermore, MDSCs participate in the proliferation, migration, EMT, and resistance to apoptosis in OSCC [131]. Clinical investigations have reported that there is a correlation between the number of MDSCs with poor survival, tumor recurrence, and metastasis [132, 133]. It has been uncovered that the release of some factors by SCC participates in the recruitment and infiltration of MDSCs. IL-8, CCL2, and CSF-1 are well-known secreted molecules by SCC that trigger the infiltration of MDSCs. As mentioned earlier, these factors are involved in the recruitment of TAMs too. The macrophage colony-stimulating factor (M-CSF) and CXCL1 are other well-known secreted molecules by SCC that stimulate the recruitment of MDSCs [134–136]. CAFs can also release CCL2, leading to the recruitment of CCR2 + MDSCs into oral cancer [137]. Stromal cells with high expression of CCL2 participate in the recruitment of CCR2 + Arg1 + MDSCs while inhibiting CCR2 in stromal cells is associated with a significant reduction in the recruitment of MDSCs [138]. An animal model has displayed that induction tongue cancer in mice is associated with the expansion of Arg1 + MDSCs in the spleen and blood, leading to the recruitment of these cells into oral tumors [139]. A clinical investigation has also reported that the infiltration of MDSCs can be involved in the progression of premalignant lesions to OSCC [140]. These findings show that MDSCs are involved in both early development and later stages of oral tumor progression and invasion.

MDSCs have several direct interactions with oral cancer cells. The release of chemokines and cytokines by oral cancer cells not only induces the recruitment of MDSCs but also stimulates the expansion of MDSCs. The SCC cells can release IL-1α, IL-6, IL-8, and granulocyte-macrophage colony-stimulating factor (GM-CSF) [141]. Some released factors such as GM-CSF can induce the differentiation of hematopoietic progenitor cells into MDSCs [138]. The mentioned cytokines can enhance the immunosuppressive effects of MDSCs. These cytokines increase the expression and phosphorylation of some key regulators such as STAT3 in MDSCs and monocytes [142, 143]. Phosphorylation of STAT3 in monocytes can stimulate the differentiation of these cells into MDSCs [144]. It has been discovered that the expression of arginase-I in MDSCs obtained from patients with HNSCC correlates with the phosphorylation of STAT3. Phosphorylated STAT3 in MDSCs can bind to the arginase-I promotor, leading to the regulation of arginase-I and enhancing the immunosuppressive effects of MDSCs [145]. Semaphorin-4D (SEMA4D) is another important secretion by HNSCC that participates in the differentiation of myeloid cells into MDSCs. It has been revealed that SEMA4D not only increases the number of MDSCs but also reduces the number and activity of CD8 + T lymphocytes in the murine model of HNSCC [146].

The inhibition of anticancer immunity is one of the most important consequences of MDSCs in oral malignancies. The inhibition of T cell trafficking is one of the key mechanisms of immune system suppression by MDSCs. It has been well demonstrated that the release of M-CSF by SCC cells stimulates the expression of CCR2 by MDSCs. The CCR2-expressing MDSCs can blunt the entry of cytotoxic CD8 + T lymphocytes into solid tumors [137]. In addition to the inhibition of lymphocyte trafficking, MDSCs in OSCC can attenuate the function of resident T lymphocytes. The release of IL-6 by OSCC and MDSCs plays a pivotal role in the regulation of the suppressive effects of MDSCs. IL-6 triggers the release of PGE2, IL-10, IL-6, IL-23, and some other suppressive molecules for T lymphocytes through the phosphorylation of STAT3. These cytokines limit the differentiation of naïve T cells into CD8 + T lymphocytes. Furthermore, the IL-6/STAT3 pathway can trigger the differentiation of CD4 + T cells into CD4 + Th17 cells in OSCC [147]. In addition to the immunosuppressive effects of MDSCs, recent experiments confirm the role of MDSCs on cancer progression and invasion. It has been revealed that MDSCs increase the proliferation of OSCC cells. Co-culture of MDSCs and OSCC cells showed a significant increase in the expression of N-cadherin and Vimentin, the well-known markers for EMT. This was associated with migration and invasion of OSCC [131]. The release of TGF-β by MDSCs can increase EMT, invasion, and resistance to therapy for different types of cancer cells [148]. However, the direct effects of MDSCs on oral cancer cells need to be more investigated.

**Fig. 3:**
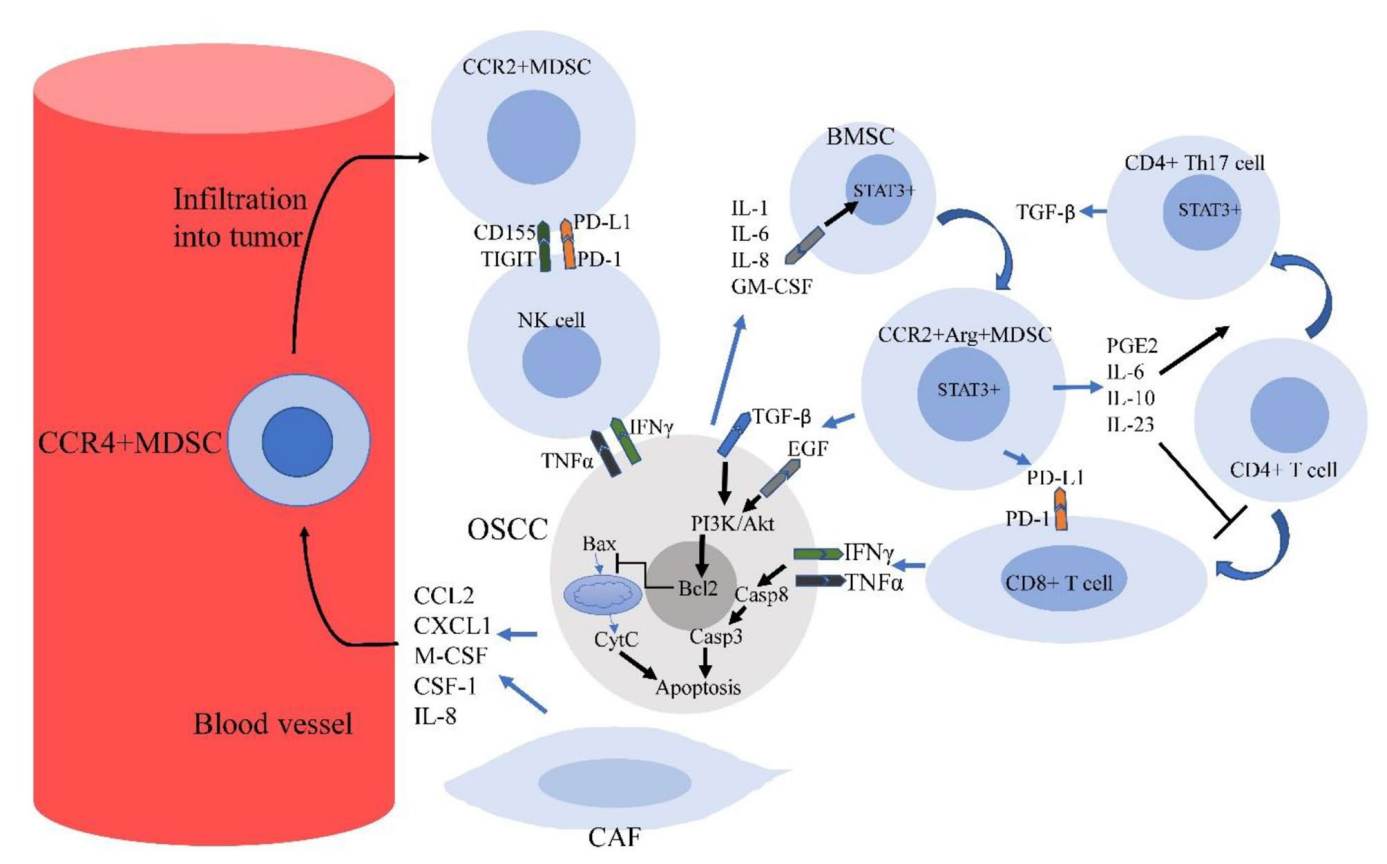
This figure illustrates the infiltration MDSCs into OSCC and interactions with cancer and anticancer immune cells in TME. The release of some factors such as CCL2, IL-8, CXCL1, M-CSF and CSF-1 by OSCC cells and CAFs stimulates the recruitment of MDSCs into the tumor. The secretions by OSCC can also induce polarization of bone marrow stem cells into MDSCs. MDSCs can exhaust NK cells and CD8 + T cells by expressing CD155 and PD-L1. Furthermore, these secretions stimulate the differentiation of CD4 + T cells into CD4 + Th17 cells while inhibiting differentiation into CD8 + T cells. The release of IFN-γ and TNF-α by NK cells and effector CD8 + T cells can stimulate apoptosis in OSCC. However, MDSCs blunt the anti-tumor activity of these cells by expressing PD-L1 and CD155. The release of growth factors by MDSCs can also inhibit apoptosis in OSCC cells. (ARG: Arginase-I; BMSC: Bone marrow stem cell; Casp: Caspase; CCR2: C-C Motif Chemokine Receptor 2; CCL-2: C-C Motif Chemokine Ligand 2; CSF-1: Colony-stimulating factor-1; CXCL1: C-X-C motif chemokine ligand 1; CytC: Cytochrome C; EGF: Epidermal growth factor; EGFR: Epidermal growth factor receptor; GM-CSF: Granulocyte-macrophage colony-stimulating factor; TGF-β: transforming growth factor-β; TIGIT: T cell immunoreceptor with Ig and ITIM domains; PD-1: Programmed death 1; PD-L1: Programmed death ligand 1; PGE2: Prostaglandin E2; PI3K: Phosphoinositide 3-kinase; M-CSF: Macrophage colony-stimulating factor; STAT: Signal transducer and activator of transcription)

The expression of some co-inhibitory proteins like PD-L1 is another mechanism of immunosuppression by MDSCs in OSCC. The cytokines such as IL-6 have a key role in the expression of PD-L1 by MDSCs. IL-6 in OSCC can induce upregulation of PD-L1 through phosphorylation of STAT3 in MDSCs. It seems that IL-6 has a more prominent role in the upregulation of PD-L1 in OSCC compared to other HNSCC cells such as pharyngeal cancer [149]. In vitro/in vivo examinations have well exhibited the role of PD-L1 + MDSCs in the suppression of CD8 + T lymphocytes and progression of OSCC. It seems that MDSCs have a higher expression of PD-L1 in the OSCC tumors compared to the spleen. This shows that MDSCs exhaust the effector CD8 + T cells within OSCC selectively [150]. CD155 is another co-inhibitory factor in OSCC-derived MDSCs. CD155 can be engaged to T cell immunoreceptor with Ig and ITIM domains (TIGIT) on the surface of NK cells and CD8 + T lymphocytes, leading to the exhaustion of these cells [151]. The role of some other co-inhibitory molecules such as T cell immunoglobulin and mucin domain-containing protein 3 (Tim-3), cytotoxic T-lymphocyte associated protein 4 (CTLA4), indoleamine 2,3-dioxygenase 1 (IDO1), and lymphocyte-activation gene 3 (LAG3) in the progression of OSCC has been confirmed [152, 153]. (Fig. 3)

It has been well exhibited that targeting of MDSCs and their suppressive molecules can boost anticancer therapy for oral tumors. MDSCs may induce potent tolerogenic responses after therapy with some drugs and also with radiotherapy. Chemotherapy and also radiotherapy can induce severe cell death and the release of some cytokines such as IL-1 and IL-6. The release of these cytokines increases resistance to therapy [154, 155]. As explained, these cytokines can increase the proliferation of MDSCs, thus increasing resistance to therapy. It has been uncovered that both radiotherapy and chemotherapy can increase the number of MDSCs [156]. The release of the mentioned cytokines and the number of MDSCs can induce the exhaustion of anticancer immunity and also stimulate the proliferation of cancer cells, as demonstrated earlier. Therefore, we need to blunt the proliferation of MDSCs and their secreted molecules to sensitize oral tumors to various types of anticancer therapy modalities.

Treatment with some drugs for reducing the number of MDSCs can attenuate tumor resistance to therapy. Treatment with ICIs is an intriguing approach for the suppression of the activity of MDSCs and boosting NK cells and CD8 + T lymphocytes against various malignancies including oral tumors [157]. The inhibition of CD155 and PD-L1 in MDSCs is an approach for targeting of MDSCs. It has been revealed that the expression of both PD-L1 and CD155 by MDSCs correlates with the stage of HNSCC tumors. Although anti-PD-L1 therapy can reduce tumor growth, it is associated with more expression of CD155 by MDSCs. Mao et al. showed that dual targeting of CD155/TIGIT and PD-L1/PD-1 in HNSCC is associated with the exhaustion of suppressive effects of MDSCs. Targeting these pathways could increase the number of effector CD8 + T cells and the release of IFN-γ, leading to more suppression of tumor growth [158]. Another experiment showed that CD155 can highly be expressed by myeloid cells and MDSCs in HNSCC tumors. The cells were obtained from patients with HNSCC. They also showed that effector CD8 + T cells have a higher expression of PD-1 and TIGIT compared to other peripheral blood mononuclear cells (PBMCs). Inhibiting the CD155/TIGIT axis led to the infiltration of CD8 + T lymphocytes and subsequent upregulation of *IL2*, *TNFα* and *IFNγ* genes by both CD4 + and CD8 + T lymphocytes. The results indicated that MDSCs and Tregs are the main suppressors of CD8 + T cells through the regulation of the CD155/TIGIT axis [159].

Tadalafil is a drug that can reduce the number of MDSCs in OSCC. A randomized clinical trial study evaluated the effect of tadalafil on oral and oropharyngeal tumors. The results demonstrated a remarkable modulation of TME following treatment with tadalafil. Administration of tadalafil caused a significant reduction of MDSCs and Tregs, which increased the infiltrated CD8 + T cells in the tumor [160]. Some other adjuvants such as natural products may be intriguing for reducing the number or exhaustion of the activity of MDSCs. For example, curcumin suppresses the phosphorylation of STAT3 in MDSCs, which leads to the exhaustion of immunosuppressive cells and the infiltration of CD8 + T lymphocytes [161].

### Regulatory T cells and oral cancers

Tregs play a substantial role in the suppression of immune responses by NK and CD8 + T cells. Forkhead box P3 (FOXP3) is the main marker of immunosuppressive Tregs [162]. The differentiation of CD4 + T cells into Tregs can be triggered by TGF-β. However, the proliferation of Tregs can be inhibited following the release of IFN-γ [163]. An increase in the peripheral number of Tregs can be detected following the development of various malignancies, including HNSCC and oral cancers [164]. Oral cancers including OSCC can induce the recruitment of Tregs by expressing some chemokines and molecules such as CCL5, CCL20, CCL22, CXCL12, and some others. Tregs may recruit into tumors like OSCC following overexpression of some receptors such as CCR6, CXCR4, CCR4, and CCR5 [165]. The expression of these markers predicts poor prognosis for patients with oral tumors [166–168]. The inhibition of these molecules also can attenuate the proliferation and transformation of OSCC [169].

Tregs have positive cross-talk with cancer and immunosuppressive cells such as MDSCs, CAFs, and TAMs. Therefore, infiltration and activation of these cells can predict poor prognosis and a weak response to therapy [170]. Tregs can render their immunosuppressive effects by releasing IL-10, TGF-β, IL-4 and IL-17, and also by regulation of some co-inhibitory molecules such as PD-L1, lymphocyte activation gene-3 (LAG3), CD69, CD73, CD39, and CD25 [171–173]. The release of IL-10 and TGF-β stimulates the differentiation of naïve CD4 + T cells into Tregs. Furthermore, these cytokines can induce the polarization of monocytes and macrophages toward TAMs. On the other hand, IL-10 and TGF-β limit the proliferation of NK cells and CD8 + T lymphocytes. In addition, the expression of PD-L1, CD39 and CD73 can exhaust the function of CD8 + T lymphocytes [174]. Although Tregs have potent immunosuppressive effects in different tumors, it seems that Tregs in oral cancers are including various subsets with different effects on immune responses. It has been reported that IL-23R + Tregs can release a high amount of IFN-γ, while IL-23R- Tregs express Foxp3 + and release a high amount of IL-10 and TGF-β in mice-bearing OSCC [175]. A clinical investigation showed various frequencies for subsets of Tregs among patients with OSCC. The findings showed that higher a frequency of IL-10 + Tregs and IL-35 + Tregs correlate with tumor growth and invasion. However, no correlation was observed for tumor stage and IFN-γ + Tregs [176]. Another study showed that CCR4+ (not CCR4-) Tregs have potent immunosuppressive effects. Therefore, it seems that CCR4 + Tregs (not total Tregs) have a correlation with poor survival of OSCC patients [177].

Because of the distinct effects of Treg subsets, these cells may cause controversial effects on invasion and also the response of oral tumors to therapy. A clinical investigation revealed that infiltrated Tregs in tonsillar SCC can predict poor prognosis. The findings from this study showed that infiltration of Foxp3 + Tregs in the tumor can predict poor disease-free survival for these patients [178]. Another study by Hayashi et al. suggested that tumor-infiltrating Foxp3 + Tregs can predict poor prognosis for OSCC patients. In this study, patients with human papillomavirus (HPV) infection and OSCC were selected for evaluation of overall and 5-year survival. The findings suggested that enrichment of Tregs in OSCC is associated with poor prognosis for both overall and 5-year survival [179]. Another clinical investigation also showed a correlation between the frequency of Foxp3 + Tregs and tumor stage for patients with OSCC [180]. By contrast, some other evaluations have shown that Tregs may don’t have a significant effect on immune system suppression and OSCC progression. A study investigated the role of Foxp3 + Tregs and CTLA4 + Tregs on the prognosis of OSCC. They showed that Foxp3 + Tregs may cause anticancer effects in patients with OSCC. In vitro and in vivo evaluations showed that CTLA4 + Tregs attenuate the function of Foxp3 + Tregs. Furthermore, some other clinical investigations suggest Foxp3 + Tregs have a positive correlation with higher survival and low frequency of metastasis in patients with OSCC [181].

Emerging findings indicate that Tregs have different immunoregulatory effects in OSCC. It seems that different subsets of Tregs may cause various effects on OSCC. For example, a high or low expression of immunosuppressive cytokines can affect anticancer or tumor-promoting effects of Tregs in oral cancers. Furthermore, the ratio of Tregs to other cells such as CD8 + T lymphocytes should be considered for different patients. A study suggested that the ratio of CD8+/FoxP3 + is a better marker for the prognosis of OSCC [182]. In addition, the role of other infective agents such as bacteria and fungi in immune responses and also the possible anti-inflammatory effects of Tregs on these responses should be considered for patients with OSCC [165]. The effects of various subsets of Tregs on immune responses in OSCC need to be evaluated in experimental and clinical studies. Further studies also need to investigate the effects of different subsets of Tregs on the proliferation and resistance to therapy for oral cancer cells.

Although the function of Tregs in OSCC is complicated, it seems that the inhibition of some released molecules by Tregs can suppress tumor growth. Anticancer drugs and also some adjuvants may be able to modulate the recruitment of Tregs and also their released molecules within oral tumors. Tregs have higher resistance to the toxic effects of ionizing radiation and chemotherapy drugs. Therefore, radiotherapy or chemotherapy drugs with high toxicity may cause an increase in the ratio of Tregs/CD8 + T cells [183]. In addition, chemotherapy and radiotherapy may stimulate the release of some molecules to induce the recruitment or activity of Tregs [184]. However, these effects may be different for various chemotherapy agents. OSCC and Tregs have positive cross-talk with each other during radiotherapy, chemotherapy, and immunotherapy, which may reduce the efficacy of anticancer drugs or ionizing radiation [185]. Therefore, using combination modalities or some specific inhibitors during cancer therapy may be useful for depleting Tregs and reducing their secreted molecules [186]. Exposure of HNSCC to ionizing radiation has shown an increase in the expression of CCL2 by cancer cells, leading to the recruitment of CCR2 + Tregs into the tumor. Furthermore, the release of TNF-α following tumor irradiation may stimulate the activity of Tregs [187].

TGF-β is one of the key released growth factors by infiltrated Tregs in OSCC [188]. Exposure of Tregs to ionizing radiation or some anticancer drugs may cause more release of TGF-β by Tregs [189]. It has been shown that the expression of TGFβ1 by some cells such as Foxp3 + Tregs in OSCC is associated with a reduction in the recruitment and proliferation of T lymphocytes. The inhibition of TGF-β expression and release by Tregs can be suggested for inhibiting different solid tumors such as oral malignancies. The inhibition of TGFβ1 can increase the infiltration of T lymphocytes and improves the anticancer potency of these cells against OSCC cells [190]. Although radiotherapy may trigger the expression and release of TGF-β, using some adjuvants or some specific chemotherapy drugs can induce immunogenic responses, which may improve the ratio of CD8 + T cells to immunosuppressive cells such as Tregs [191]. It has been well demonstrated that the expression of immunosuppressive markers such as Foxp3 and TGFβ1 has a direct relation with infiltrated Tregs in the tumors of patients that underwent radiotherapy [192].

Chemotherapy drugs such as 5-FU and also hypofractionated radiotherapy can induce immunogenic cell death [193]. Treatment with these drugs may induce immunogenic responses in oral malignancies, which lead to more infiltration of T lymphocytes and suppression of immunosuppressive cells like Tregs. A study showed that using a combination of radiotherapy and 5-FU can repress the infiltration of Tregs. This study first used OK-432 to evaluate immune responses in OSCC. OK-432 is an anticancer drug for HNSCC. It acts through the stimulation of NK cells. However, the release of some soluble factors by OSCC can blunt the release of anticancer cytokines following treatment with OK-432 [194]. It has been shown that treatment of OSCC with OK-432 causes overexpression of Foxp3 + and the release of IL-10 and TGF-β. In addition, a reduction in the release of Th1 cytokines was reported. However, treatment with 5-FU and ionizing radiation reversed the expression of Foxp3 + and the release of Th2 cytokines. Combination therapy with radiation and 5-FU also reduced the expression of suppressor of cytokine signaling 1 (SOCS1) and SOCS3, the key inducers of Foxp3 + Tregs in solid tumors. The findings also indicated that a reduction in the immunosuppressive cytokines following treatment with radiation and 5-FU can improve the activity of NK cells and T cells by enhancing the release of IL-12 and IFN-γ [195]. Modulating the function of Tregs is an intriguing approach for overcoming resistance to therapy. Radioresistant OSCC may show low response to radioimmunotherapy. However, depletion of Tregs with anti-CD25 has been shown to increase the efficacy of radioimmunotherapy. It has been revealed that depletion of Tregs in combination with anti-CD137 (for activation of DCs) and radioimmunotherapy can eradicate OSCC tumors [196].

### Tumor-associated neutrophils and oral cancers

TANs contribute to the progression of oral tumors by regulating some mechanisms such as inhibiting immune responses in CD4 + and CD8 + T cells, and also by inducing angiogenesis and metastasis. Investigations from oral tumor samples confirm a direct relationship between the infiltration of neutrophils and poor prognosis for patients with oral cancers [197]. The ratio of neutrophils to lymphocytes in oral cancers also correlates with poor prognosis and lower survival of patients [198]. However, neutrophils and TANs have shown various effects in favor or unfavor of tumor progression. TANs may induce adaptive immune system responses and also trigger the activity of T lymphocytes by stimulating the antigen process by APCs [199]. Therefore, it has been suggested that neutrophils in the tumor may act in favor of tumor suppression or progression, depending on their interactions with cancer cells and the TME [200]. Taken together, targeting neutrophils in oral tumors isn’t an interesting therapeutic target compared to other immune cells and targets within TME.

### B lymphocytes in oral cancer

B cells can accumulate within tertiary lymphoid structures (TLSs). TLSs in solid tumors can facilitate the entrance of immune cells into tumors [201]. The expansion of B lymphocytes is associated with controversial consequences for various tumors. Some studies suggested that TLSs can predict more survival of patients with cancer such as gastric cancer [202, 203]. However, TLSs may predict metastasis and higher grade of cancers [203]. Recently, some studies suggested that TLSs and infiltration of B lymphocytes are associated with a better prognosis for patients with OSCC [204–207]. However, a clinical investigation suggested that peritumoral B cells can predict the invasion and metastasis of OSCC [208]. As TLSs have been shown to contribute to the response of tumors to anticancer drugs such as ICIs [209], understanding the role of B lymphocytes in anti-tumor immunity is an intriguing issue. B cells can act against cancer cells after differentiation into memory B-cells and also plasma cells. It seems that B cells have a key role in the activation and expansion of T lymphocytes. Antigen presentation by B lymphocytes can augment the expansion and memory formation of T lymphocytes following priming by DCs [210]. B lymphocytes also stimulate the activity and expansion of CD8 + T lymphocytes by producing antibodies, cytokines, and chemokines, and also by the formation of memory for these cells [211]. B lymphocytes may kill cancer cells directly by releasing pro-apoptosis molecules such as FasL [212].

The role of B lymphocytes in oral tumors is little known. However, some studies have investigated the possible interactions of B lymphocytes with other cells within oral tumors. It has been reported that the abundance of B lymphocytes is associated with more release of CXCL9 production and infiltration of CD8 + T lymphocytes in oropharyngeal SCC. The findings indicated that infiltrating memory B cells are the main type of B cells within the tumor, however, no accumulation of B regulatory cells (Bregs) was observed in oropharyngeal SCC. The findings also showed that B lymphocytes exert their anticancer effects by increasing the survival of CD4 + and CD8 + T lymphocytes [213]. In contrast to this study, another study by Zhou et al. showed that Bregs in tongue SCC can trigger the differentiation of CD4 + T lymphocytes into Tregs [214]. In total, it seems that although CD20 + B cells have anti-tumor activity in oral cancers, Bregs in some types of these malignancies may be involved in immunosuppression. Similar to other subsets of lymphocytes, B cells have a high sensitivity to anticancer drugs and ionizing radiation. Therefore, a decline in the number of B and T cells in higher stages of oral tumors and also after chemotherapy or radiotherapy is predictable. It has been reported that a higher ratio of Bax/Bcl-2 in lymphocytes is responsible for the higher sensitivity of B and T cells to apoptosis [215].

### CD4 + T lymphocytes in oral cancer

CD4 + T cells include various subsets such as CD4 + Foxp3 + Tregs, CD4 + Th1 cells, CD4 + Th2 cells, CD4 + Th17 cells, CD4 + memory T cells, and some others. Naïve CD4 + T cells can differentiate into each type of the mentioned cells following exposure to antigens and the released cytokines within the tumor milieu [216, 217]. As explained, CD4 + Foxp3 + Tregs are potent immunosuppressive cells that may help tumor progression. Because of different subsets with various interactions, studies have reported controversial effects on the role of CD4 + T cells in oral tumors [218]. CD4 + Th1 cells stimulate inflammatory responses by releasing inflammatory cytokines such as IFN-γ and TNF-α. However, CD4 + Th2 cells suppress the activity of inflammatory cells such as NK cells, CD8 + T lymphocytes, and CD4 + Th2 cells by releasing IL-4, IL-10, IL-13, and TGF-β. The anti-tumor effects of CD4 + Th1 and pro-tumor effects of CD4 + Th2 cells have been confirmed in several studies. It has been reported that a higher number of CD4 + Th1 is associated with tumor suppression while a higher number of CD4 + Th2 cells is associated with oral tumor growth [219]. CD4 + memory T cells can release some anti-tumor molecules such as IFN-γ, TNF-α, and Granzyme B. Therefore, a high frequency of these cells in the TME is associated with better survival of patients with cancer [220, 221]. Recently, a study confirmed that tumor-infiltrated memory CD4 + T cells correlate with better prognosis in patients with oral cancer [222].

It has been reported that patients with OSCC have a higher number of CD4 + Th17 cells in the tumor and blood, which may promote tumor growth [147]. CD4 + Th17 cells may cause different consequences for various tumors [218]. These cells may release tumor-promoting cytokines and pro-angiogenesis molecules such as VEGF. On the other hand, CD4 + Th17 cells can induce inflammatory responses by inducing the recruitment of CD8 + T lymphocytes. These various effects are associated with different effects for different tumor types [223]. Differentiation into CD4 + Th17 can be triggered by IL-6 and TGF-β. CAFs can facilitate the differentiation of CD4 + Th17 cells by releasing these cytokines. On the other hand, the release of IL-17 by CD4 + Th17 cells can activate CAFs [224]. This positive cross-talk between CAFs and CD4 + Th17 cells can promote oral tumor growth and reduce response to therapy. A higher number of CD4 + Th17 cells in oral cavity tumors may also predict resistance to immunotherapy [225]. Because of the different consequences of CD4 + T cells in tumors, most studies didn’t focus on targeting these cells for oral cancer therapy. However, the existence of these cells may be crucial for response to anticancer drugs such as tumor vaccines [226]. Furthermore, pharmacological targeting of Th2-mediated immunity may increase the response of tumors to immunotherapy [227]. However, this needs to be investigated for oral tumors.

### CD8 + T lymphocytes in oral cancer

CD8 + T lymphocytes are the key anti-tumor immune cells in the TME. The cytotoxic function of CD8 + T lymphocytes needs antigen presentation by APCs. DCs can carry cancer antigens toward CD8 + T lymphocytes which leads to the maturation and activation of these cells [228]. The infiltrated CD8 + T lymphocytes play a pivotal role in the response of various tumors to anticancer drugs such as ICIs and chemotherapy [229]. Clinical studies have confirmed the positive effects of CD8 + T lymphocytes on the prognosis of various solid tumors including oral malignancies [230]. The main released molecules by CD8 + T lymphocytes are consisting of inflammatory cytokines such as IFN-γ and TNF-α, and also lytic enzymes such as Granzyme B and perforin. It has been reported that some CD8 + T cells in oral tumors express CD103. The infiltration and activity of CD103 + CD8 + T cells in OSCC predict a good prognosis and also a better response to immunotherapy [231, 232]. The release of Th1 cytokines by CD4 + T cells and also the presence of IFN-γ can stimulate differentiation and activation of CD8 + T cells. Furthermore, DCs can improve the cytotoxic activity of CD8 + T lymphocytes by releasing some cytokines such as IL-12 [233]. On the other hand, the regulation of co-inhibitory molecules such as PD-L1, Tim-3, TIGIT, and CTLA4 by cancer cells, Tregs, TAMs, DCs, and MDSCs can exhaust the anticancer potency of cytotoxic CD8 + T lymphocytes [234].

The presence and activity of CD8 + T lymphocytes are crucial for inhibiting tumor growth and also oral cancer response to therapy. Investigations in clinical specimens show a reduction in the number of CD8 + T lymphocytes during the invasion of OSCC. In addition, tumors with a low frequency of CD8 + T lymphocytes have a poor response to immunotherapy [235]. The stimulation of the cytotoxic activity of CD8 + T lymphocytes or inhibiting the co-inhibitory mediators in these cells can increase the efficacy of therapy for OSCC. The activity and maturation of CD8 + T lymphocytes can be induced by cancer antigen presentation. A reduction in the presentation and expression of OSCC tumor antigens reduces the infiltration and cytotoxic activity of CD8 + T lymphocytes [236]. In vitro evaluations have confirmed that stimulation of CD8 + T lymphocytes by antigens from patients with OSCC enhances the anti-tumor potency of CD8 + T lymphocytes [237]. Stimulation of CD8 + T lymphocytes using tumor antigens can be suggested to suppress OSCC. Some vaccines have been developed to suppress oral cancers by stimulating the activity of CD8 + T lymphocytes in experimental tumor models [238, 239]. However, it seems that vaccines have a long way to be approved for effective oral cancer therapy. Treatment with radiotherapy is another approach for inducing antigen expression by cancer cells and activation of CD8 + T lymphocytes. Radiotherapy can upregulate the expression of specific antigens by cancer cells that help APCs for the detection of cancer cells and stimulation of CD8 + T lymphocytes [184]. In addition, immunogenic cell death after radiotherapy can stimulate anti-tumor immunity. Hypofractionated radiotherapy techniques such as SBRT can stimulate immunologic cell death and inflammatory responses, which lead to the release of damage-associate molecular patterns (DAMPs), maturation of DCs, and finally activation of effector CD8 + T lymphocytes [240]. Infiltration of CD8 + T lymphocytes following radiotherapy has been approved for patients with OSCC [241]. A similar effect can be observed for some specific chemotherapy drugs. Chemotherapy drugs that can induce immunogenic cell death like necrosis or pyroptosis may be useful for stimulating anticancer responses by CD8 + T lymphocytes. For example, the induction of pyroptosis in OSCC is positively correlated with the infiltration of CD8 + T lymphocytes [242]. Despite the beneficial effects of radiotherapy and some immunogenic drugs, the exposure of Tregs, DCs, TAMs, and also cancer cells to some DAMPs such as adenosine lead to overexpression of some co-inhibitory molecules such as Tim-3, CTLA4, TIGIT, and PD-L1 [243]. Therefore, treatment with combination modalities is more effective for inducing prolonged anticancer immunity by CD8 + T lymphocytes. Targeting DAMPs like adenosine is an intriguing approach for remodeling TME. Adenosine can stimulate the expression of Foxp3 in Tregs. In addition, exposure of Tregs to adenosine is associated with the release of TGF-β, which lead to the exhaustion of CD8 + T lymphocytes. It has been exhibited that the inhibition of adenosine A2A receptor in tongue SCC is associated with the activation of CD8 + T lymphocytes and a reduction in the number of Foxp3 + Tregs, leading to tumor repression [244].

Immune checkpoints are the key co-inhibitory molecules for CD8 + T lymphocytes. Therefore, a large number of experimental and clinical studies have investigated the targeting of different immune checkpoints for inhibiting oral malignancies [245]. The expression of PD-1 by cytotoxic CD8 + T lymphocytes plays a key role in the exhaustion of these cells. The engagement of PD-L1 to PD-1 is associated with apoptosis and inactivation of cytotoxic CD8 + T lymphocytes [246]. It has been uncovered that overexpression of PD-L1 in OSCC is associated with a reduction in the infiltrated CD8 + T lymphocytes [247]. The suppressive effect of PD-L1 + Tregs on the infiltrated CD8 + T lymphocytes in OSCC has been confirmed [248]. Overexpression of PD-L1 in OSCC can be regulated by some mediators. It has been uncovered that the release of IFN-γ by cytotoxic CD8 + T lymphocytes is a substantial inducer of PD-L1 in cancer cells and also other immunosuppressive cells in TME. IFN-γ can stimulate the phosphorylation of STAT3 and activation of NF-κB, leading to overexpression of PD-L1 [249]. Some other mediators can stimulate the regulation of PD-L1 and the exhaustion of CD8 + T lymphocytes. Circular RNA (CircRNA) keratin 1 is a non-coding RNA that regulates the expression of PD-L1 by targeting miR-495-3p. It has been revealed that aberrant regulation of CircRNA keratin 1 in OSCC participates in the overexpression of PD-L1 by sponging miR-495-3p [250]. miR-495-3p works as a tumor suppressor, therefore, its inhibition in OSCC is associated with tumor progression and invasion [251]. TIGIT and CTLA4 are other co-inhibitory regulators of cytotoxic CD8 + T lymphocytes. Evaluating the specimens from patients with OSCC shows that CD8 + T cells have a higher expression of TIGIT compared to CD4 + T cells. Overexpression of TIGIT is associated with a lower release of IFN-γ and a higher release of IL-10 by CD4 + T lymphocytes. However, in vitro evaluations confirmed that the inhibition of TIGIT augments the release of anti-tumor cytokines by CD8 + T cells [252].

Tim-3 overexpression is another mechanism for the attenuation of CD8 + T lymphocytes in OSCC. Similar to other co-inhibitory mediators, Tim-3 can be expressed by immunosuppressive cells such as Tregs and also by DCs. It has been shown that the expression of Gal-9 by CD4 + T cells stimulates the overexpression of Tim-3 by monocytes. These changes are associated with the exhaustion of effector CD8 + T lymphocytes and a reduction in the secretion of IFN-γ [253]. miR-545-5p is an inhibitor of Tim-3. However, its regulation in OSCC can be suppressed by colorectal neoplasia differentially expressed (CRNDE). CRNDE is a lncRNA that can be overexpressed in some malignancies. The regulation of CRNDE in OSCC is associated with the exhaustion of effector CD8 + T lymphocytes and the downregulation of IFN-γ by these cells. However, the inhibition of CRNDE in naïve CD8 + T cells can enhance the cytotoxicity of effector CD8 + T lymphocytes against cancer cells by inducing miR-545-5p and downregulating Tim-3 [254].

The expression of some other molecules can also attenuate the activity of CD8 + T lymphocytes. TGF-β is a potent inhibitor of CD8 + T lymphocytes. Tregs and TAMs are the main sources of TGF-β in OSCC. However, cancer cells also can express a high amount of TGF-β in the advanced stages of the tumor [255]. The expression of TGFβ1 in OSCC has a direct relation with the ratio of Tregs/T cells. Increasing the expression of TGFβ1 in OSCC not only reduces CD8 + T lymphocyte numbers but also attenuates the release of anticancer cytokines by effector CD8 + T lymphocytes [190]. Blockade of Tregs or some other immunosuppressive cells or molecules is an intriguing approach for improving the function of effector CD8 + T lymphocytes. Ablation of Tregs in OSCC has been shown to increase the infiltration of CD8 + T lymphocytes [256]. As Tregs are the main sources of immunosuppressive molecules for CD8 + T lymphocytes, an increase in the proliferation and recruitment of CD8 + T lymphocytes following the ablation of Tregs is predictable.

### Natural killer cells in oral cancer

NK cells are among the most important players in innate immunity. Some anticancer cytokines such as IFN-γ, IL-2, and IL-12 can stimulate the differentiation of CD4 + T cells into NK cells [257]. By contrast to CD8 + T lymphocytes, NK cells can lysis or induce apoptosis in malignant cells without the need for antigen presentation by APCs [258]. NK cells can differentiate normal cells from cancer cells through the detection of some specific antigens such as major histocompatibility complex (MHC). MHC I and MHC II can normally be expressed by typical cells. However, cancer cells express a lower amount of these proteins. Therefore, NK cells attack abnormal cells with unusual MHC I and MHC II [259]. The main released molecules by NK cells are consisting of TNF-α, IFN-γ, IL-2, IL-8, and also lytic granules consisting of perforin and granzyme B. The mentioned cytokines not only stimulate apoptosis in cancer cells but also can promote the differentiation of CD4 + T cells into anticancer immune cells. Furthermore, the released granules can cause the lysis of cancer cells [260]. In TME, cancer cells and immunosuppressive cells such as MDSCs, Tregs, and TAMs can potently suppress the activity of NK cells by releasing Th2 cytokines and also the regulation of co-inhibitory molecules such as CD38, CD80/86, CD155, PD-L1/2, Gal9, and some others [261, 262]. CD80/86 can bind to CTLA4, leading to the exhaustion of NK cells. The activity of these cells can also be exhausted by other engagements such as CD155-TIGIT, PD-L1/2-PD-1, Gal9-Tim-3, and others [263]. The positive prognosis of NK cells in various tumors like oral cancers has been observed [264]. Furthermore, infiltration and improvement of NK cell activity can increase the survival of patients with OSCC [264].

The activity of NK cells can be induced or inhibited by several molecules in NK cells and cancer or immunosuppressive cells. The natural killer group 2, member D (NKG2D) is among the most important inducers of NK cell activity. Furthermore, the expression of some other receptors such as TLR2 is crucial for regulating the activity of NK cells [265]. Some ligands such as MHC class I-related chain family (MICA/B) and UL16 binding protein (ULBP)1 and 2 expressed by cancer cells can activate NK cells by binding to NKG2D on the surface of NK cells. Although the expression of NKG2D ligands by cancer cells can activate the cytotoxicity of NK cells, an increase in the soluble level of these ligands can shed on NKG2D, leading to immune evasion [266]. Immune evasion of oral cavity cancers is correlated with serum levels of NKG2D ligands. Therefore, inhibiting these ligands can improve the activity of NK cells and the response of tumors to immunotherapy [267]. Depletion of these ligands recovers the release of TNF-α and IFN-γ by NK cells. Restoring the activity of NK cells by depleting NKG2D ligands can improve the response of HNSCC to other anticancer drugs like cetuximab [268]. Activation of NK cells by a TLR2 ligand has also been shown to enhance the anticancer efficacy of cetuximab against tongue SCC [269].

The infiltration and activity of NK cells are very crucial for the response of tumors to different anticancer modalities. Unfortunately, radiotherapy and classic chemotherapy drugs can drop the level of NK cells, thus attenuating anticancer responses by these cells [270]. In addition, exposure of tumors to ionizing radiation or chemotherapy causes overexpression of co-inhibitory molecules by cancer and immunosuppressive cells. Increased expression of co-inhibitory molecules such as CD155, Tim-3 and PD-L1/2 suppresses the infiltration of NK cells [271, 272]. Therefore, some experiments have been performed to assess the therapeutic efficacy of a combination of NK cell immunotherapy with other anticancer agents. Targeting co-inhibitory molecules is an intriguing approach for increasing the number of NK cells in solid tumors [273]. An increase in the anti-tumor activity of NK cells following treatment with both radiotherapy and ICIs has been reported by Patin et al. [274]. The findings by these authors showed that radiotherapy of OSCC stimulates the regulation of TIGIT by NK cells. However, selective inhibition of TIGIT improved anti-tumor immunity in murine models OSCC [274]. Although the role of PD-L1/PD-1 blockade on NK cells is controversial, some studies have reported that targeting this axis can be proposed for boosting the activity of NK cells [275, 276]. Targeting Tim-3 for boosting NK cell activity is under investigation in clinical trial studies. Primary results are promising [277]. However, it needs to be investigated for different tumors such as oral malignancies.

**Fig. 4:**
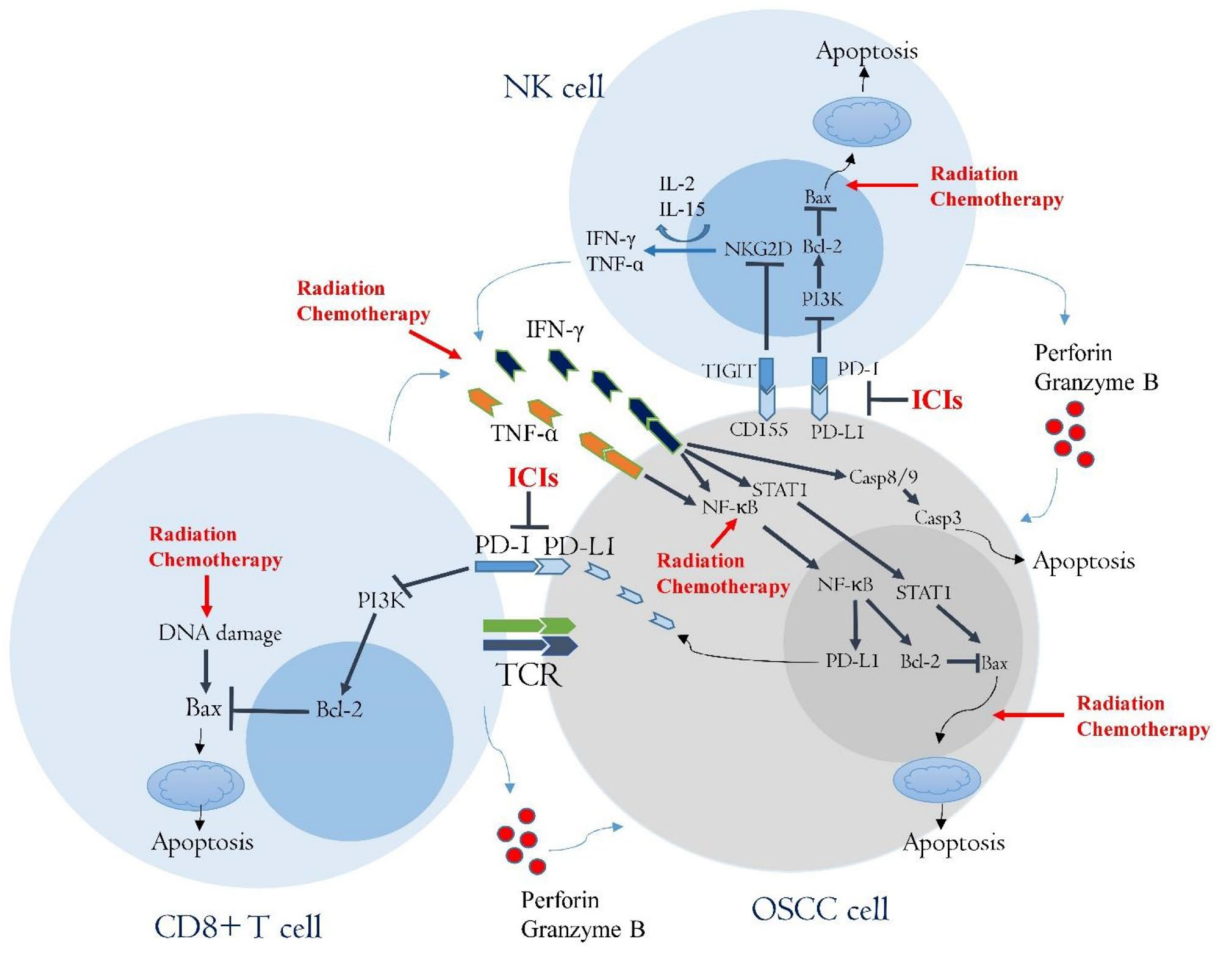
This figure illustrates the interactions of OSCC with NK cells and CD8 + T lymphocytes. The detection of cancer cells by NK cells and CD8 + T lymphocytes leads to the release of lytic enzymes and also anticancer cytokines, including TNF-α and IFN-γ. These cytokines not only induce apoptosis in OSCC cells but also trigger the expression of PD-L1. PD-L1 can bind to PD-1, leading to the exhaustion of NK cells and CD8 + T cells. The expression of CD155 by OSCC cells has a similar consequence. Radiotherapy and chemotherapy can also boost the release of TNF-α and IFN-γ by inducing immunogenic cell death in TME. However, exposure of NK cells and lymphocytes to radiation or chemotherapy drugs can induce apoptosis in these cells. Inhibiting the PD-L1-PD-1 axis or the CD155-TIGIT axis can prevent the exhaustion of NK cells and CD8 + T lymphocytes. In addition, treatment with IL-2 or IL-15 can increase the expression of NKG2D and the release of anticancer cytokines. (Casp: Caspase; ICIs: Immune checkpoint inhibitors; NKG2D: natural killer group 2, member D; TCR: T cell receptor; TIGIT: T cell immunoreceptor with Ig and ITIM domains; NF-κB: nuclear factor-κB; PD-1: Programmed death 1; PD-L1: Programmed death ligand 1; PI3K: Phosphoinositide 3-kinase; STAT: Signal transducer and activator of transcription)

Cytokine therapy is another approach for boosting NK cells. IL-2 is the best-known cytokine for boosting anti-tumor immunity by NK cells. However, IL-15 can also stimulate the activity of NK cells against malignant cells. It has been revealed that these cytokines stimulate the expression of NKG2D in NK cells, leading to the release of lytic enzymes and anticancer cytokines [278]. Treatment with IL-2 has been shown to boost the release of FMS-related tyrosine kinase 3 ligand (Flt3L) by NK cells. Flt3L stimulates antigen presentation by DCs, leading to the maturation of CD8 + T lymphocytes. This effect of IL-2 has been reported as a mechanism for a synergistic effect of IL-2 and radiotherapy against oral tumors [279]. As immunosuppressive cells are the key suppressors of NK cells, targeting some cells such as MDSCs can increase the infiltration of NK cells and boost their cytotoxicity against cancer cells. Although direct boosting of NK cells by IL-2 can stimulate the lysis of MDSCs by NK cells [276], some studies have tried to boost the infiltration of NK cells by inhibiting MDSCs in oral tumors. For example, targeting CXCR1/2 in murine oral tumors can inhibit the infiltration of MDSCs, leading to more accumulation and activity of NK cells [280]. The inhibition of immunosuppressive cells such as TAMs, MDSCs, Tregs, and CAFs can boost the infiltration and activity of NK cells, as explained earlier. In the previous section, the approaches for targeting these cells have been explained. Furthermore, as NK cells have positive cross-talk with CD8 + T lymphocytes, infiltration of CD8 + T cells can trigger the activity of NK cells, and vice versa (Fig. 4).

## Hypoxia and HIF-1α in oral cancer resistance to therapy

Low oxygen and glucose concentration is a typical event in solid tumors. This occurs in solid tumors because of incomplete vascular development during the extraordinary proliferation of cancer cells [281]. Low oxygen and glucose concentrations change the metabolism of cancer cells. Triggering autophagy and glycolysis in a low glucose and oxygen condition increases the resistance of cancer cells to therapy [282]. Activation of hypoxia-inducible factors (HIFs) is one of the major consequences of hypoxia in the tumor. In a normal level of oxygen concentration, prolyl hydroxylase domain proteins (PHDs) degrade HIF-1α. However, low concentrations of oxygen inactivate PHDs, leading to the activation of HIF-1α [283]. Although hypoxia is the pivotal inducer of HIF-1α, some other changes in pre-malignant and malignant lesions can induce regulation of HIF-1α. It seems that overexpression of HIF-1α by pre-malignant or infiltrated cells plays a pivotal role in the development of OSCC. The positive cross-talk of chronic inflammation with oncogenesis has been demonstrated by several studies. Activation of HIF-1α by inflammatory cytokines plays a key role in oral transformation and tumorigenesis. IL-1 can stimulate HIF-1α in oral lesions through the NF-κB/COX-2 axis [284]. Upregulation of HIF-α and inflammatory cytokines may be involved in the tumorigenesis in the oral cavity and can be proposed as biomarkers for oral malignancies [285, 286]. HIF-1α may be involved in the early development of HPV-induced OSCC [287]. An aberrant increase in the activity of HIF-1α following polymorphism in HIF-1α or its upstream genes can also be involved in the development of OSCC [288].

After tumor development, HIF-1α can also be induced by hypoxia and inflammatory mediators [289, 290]. Hypoxia can be induced following the occlusion of vessels by tumor cells or insufficient diffusion of oxygen into tumors [291]. Tumor stiffness is another player in hypoxia. Accumulation of collagen and fibrin enhances tumor stiffness. CAFs are the main sources of collagen and are the key player in OSCC tumor stiffness [292]. All the mentioned changes in oral tumors enhance the regulation of HIF-1α. The regulation of HIF-1α in patients with OSCC can also be induced by the B7 homolog 3 protein (B7-H3) through PI3K/Akt/mTOR pathway. Activation of HIF-1α by this immune checkpoint regulates glycolysis in OSCC and increases glucose uptake by cancer cells. These consequences of hypoxia reduce the available glucose of CD8 + T lymphocytes [293]. It has been revealed that hypoxia reduces the expansion and cytotoxicity of T lymphocytes by inducing the regulation of PD-L1 in OSCC [294]. Hypoxia can also stimulate the recruitment of MDSCs, which leads to the exhaustion of NK cells and CD8 + T cells. In addition, the generation of lactate in hypoxic TME stimulates the polarization of macrophages toward TAMs [295]. A correlation between the expression of HIF-1α and infiltration of TAMs has been reported in patients with OSCC [296]. CAFs are other cells in TME that increase stemness and chemoresistance of oral cancer cells during hypoxia by releasing some specific molecules such as serglycin and fibroblast activation protein (FAP) [297].

Changes in the metabolism of cancer cells following overexpression of HIF-1α reduce oxygen consumption to adapt cancer cells to low oxygen conditions [282]. Activation of HIF-1α is associated with overexpression of pro-angiogenesis molecules such as VEGF. Cancer cells and also some other cells such as CAFs, MDSCs, TAMs, and Tregs can release VEGF during hypoxia [298]. Overexpression of HIF-1α and VEGF not only increases resistance to various anticancer drugs and radiotherapy [299] but also promotes OSCC invasion and metastasis [300–302]. HIF-1α induces overexpression of miR-210-3p in OSCC. Indeed, miR-210-3p is known as hypoxia-inducible miRNA. miR-210-3p inhibits the expression of repulsive guidance molecule A (RGMA), a negative regulator of angiogenesis and tumor metastasis [303, 304]. Upregulation of HIF-1α/miR-210-3p and downregulation of RGMA in OSCC is known as a mechanism for the proliferation, invasion, and migration of cancer cells [305]. Stimulation of the Kv3.4 channel in OSCC is another suggested mechanism for inducing invasion by HIF-1α [306]. Upregulation of this channel is associated with overexpression of vimentin, which promotes EMT and migration of cancer cells [307, 308]. Histone deacetylase 2 (HDAC2) and MIR31HG (a LncRNA) are other co-activators of HIF-1α in OSCC [309, 310].

Hypoxia and overexpression of HIF-1α participate in the resistance of OSCC to chemotherapy, radiotherapy, and immunotherapy [311]. In addition, radiation and some chemotherapy drugs can trigger the regulation of HIF-1α. Therefore, inhibition of HIF-1α can be suggested for reducing oral cancer resistance to therapy [312]. It has been revealed that inhibiting HIF-1α can stimulate various mechanisms of cell death such as apoptosis and ferroptosis in OSCC [313]. Some experiments have been conducted to sensitize oral cancer cells to radiation or drugs by inhibiting HIF-1α. The inhibition of HIF-1α using a siRNA has been shown to increase apoptosis in OSCC [314] and sensitize these cells to ionizing radiation or 5-FU [311]. Some anti-HIF-1α drugs have been developed for the sensitization of solid tumors to chemotherapy or radiotherapy. PX-478 as a HIF-1α inhibitor has been shown to sensitize various solid tumors such as HNSCC to chemotherapy and ionizing radiation [315–317]. In phase I clinical trial study, PX-478 has also shown well toleration with an observable reduction in the HIF-1α [318]. Recently, a study suggested that HIF-1α inhibition by PX-478 can sensitize solid tumors to ICIs [319]. YC-1 is another HIF-1α inhibitor that has been shown to sensitize OSCC to various anticancer agents such as radiation, chemotherapy, and hyperthermia [320, 321]. Experiments for exploring HIF-1α inhibitors for solid tumors including oral malignancies are ongoing. Some other adjuvants such as metformin and glucosamine have been shown to increase apoptosis in oral cancer cells and sensitize these cells to chemotherapy by inhibiting HIF-1α [322, 323].

## Conclusion and future perspectives

Increasing cancer incidence and mortality make it one of the most causes of death in the world. Oral cancers are among the cancers with growing incidence, especially for developing cancers and also for countries with higher consumption of alcohol and tobacco. Although early detection of cancers increases the chance of tumor eradication, side effects associated with tumor therapy can significantly affect the quality of life for surviving patients. A piece of deep knowledge about oral TME can provide new approaches to the elimination of tumors. It seems that tumor stroma and CAFs play a pivotal role in the development and invasion of oral malignancies. CAFs and stroma orchestrate a way for the recruitment of other immunosuppressive cells and also the exhaustion of CD8 + T lymphocytes and NK cells. The release of several molecules such as chemokines and cytokines by oral cancers stimulates the recruitment of monocytes, Tregs, MDSCs, stem cells, macrophages, and some others. These cells have positive cross-talk with each other and also with cancer cells. The released molecules by these cells not only stimulate the recruitment of more immunosuppressive cells but also increase the resistance of cancer cells against NK cells and CD8 + T lymphocytes. The main effects of TME on cancer cells and anticancer immune cells can be induced through some mechanisms. These mechanisms are including the recruitment of immunosuppressive cells, the expression of several co-inhibitory molecules such as PD-L1, CD155 and Tim-3, and also the release of immunosuppressive cytokines and growth factors. These interactions facilitate the proliferation of cancer cells and on the other hand, attenuate the function of anticancer immune cells. Targeting these interactions can boost the immune system and suppress tumor growth.

In recent years, some experiments have shown promising results for the suppression of oral malignancies by targeting various cells and molecules within TME. The depletion or reducing the number of immunosuppressive cells has been shown to suppress the growth and also the resistance of oral cancers to anticancer drugs. The depletion of TAMs or reducing MDSCs by antibodies or some adjuvants has been shown that not only induces apoptosis in cancer cells but also reduce the number of other immunosuppressive cells. In addition, it can boost the release of anti-tumor cytokines and lytic enzymes by NK cells and CD8 + T lymphocytes. Targeting some chemokines and molecules such as CCL2, CSF-1, CXCR4, CXCL12, CCR4, and some others have shown that can help to oral tumor suppression by reducing the number of immunosuppressive cells. However, several other chemoattracts are involved in the recruitment of immunosuppressive cells that can be targeted for reducing the number of immunosuppressive cells. These cells have positive cross-talk with cancer cells and also with other immunosuppressive cells in TME mainly by releasing immunosuppressive cytokines such as IL-4, IL-10, IL-13, and TGF-β. Unfortunately, radiotherapy and chemotherapy can increase the release of these cytokines, thereby accelerating the proliferation of immunosuppressive cells. These cytokines also can attenuate the response of tumors to immunotherapy. Therefore, a reduction in the release of these cytokines using some adjuvants can be suggested for boosting anticancer immunity during therapy. The immunosuppressive effects in the hypoxic TME can be amplified because of the exhaustion of NK cells and CD8 + T lymphocytes. Some experiments have shown an increase in the sensitivity of OSCC to anticancer agents using anti-HIF-1 agents. However, this topic needs further studies in the future.

Immunotherapy for blocking co-inhibitory molecules is one of the most novel approaches for targeting various tumors including oral malignancies. Cancer cells, immunosuppressive cells, and also DCs can express co-inhibitory molecules for NK cells and CD8 + T lymphocytes. The PD-L1/PD-1 axis is the most known target for boosting anticancer immunity in various tumors like oral cancers. However, some other targets such as CTLA4 and Tim-3 are under investigation in the experimental and clinical trial studies. Blocking CD155-TIGIT is an intriguing approach for boosting the anticancer potency of NK cells. Experimental studies have shown promising results for oral cancers. A combination of ICIs for these co-inhibitory molecules in combination with other treatment modalities such as radiotherapy or receptor tyrosine kinase inhibitors has shown promising results in pre-clinical studies. Further studies in clinical trial studies need to investigate these combination modalities in clinical trial studies.

There is no confident knowledge about the interactions of some other cells in oral TME. B cells, CD4 + T cells, Tregs, and neutrophils have controversial effects on oral tumors. Tregs have potent immunosuppressive effects that can increase resistance to therapy for a wide range of malignancies. Experimental studies also indicate that Tregs play a substantial role in the exhaustion of effector CD8 + T lymphocytes. However, clinical findings suggest controversial effects for these cells. It seems that further studies need to evaluate the role of these cells in the response of different oral tumors to various anticancer therapy modalities such as radiotherapy, immunotherapy, and chemotherapy. As Tregs may have protective effects against inflammatory responses by HPV, the role of these cells in patients with HPV + and HPV- should be considered. Similar considerations should be noted for B cells and neutrophils. It seems that B cells in some oral tumors participate in the development of anticancer immunity while they may develop immunosuppressive Bregs in others. CD4 + T cells have different subsets with various interactions. However, some subsets such as CD4 + Th17 cells have different effects in favor of both tumor suppression and progression. Targeting these cells and interactions may don’t cause a favorable response by oral tumors to therapy.

## Data Availability

Not applicable.
